# Techno-economic analysis of industrial-scale fermentation for formate dehydrogenase (FDH) production

**DOI:** 10.1186/s40643-025-00985-3

**Published:** 2025-12-06

**Authors:** Julia Cunniffe, Vanessa Rondon Berrio, Cameron Hunter, Thuan Nguyen, Sonja Salmon, Nathan Crook, Amy Grunden, William Joe Sagues

**Affiliations:** 1 Department of Biological and Agricultural Engineering, North Carolina State University, 3110 Faucette Drive, Raleigh, NC 27695 USA; 2 Department of Plant and Microbial Biology, North Carolina State University, 840 Oval Drive, Raleigh, NC 27606 USA; 3 Department of Chemical and Biomolecular Engineering, North Carolina State University, 911 Partners Way, Raleigh, NC 27606 USA; 4 Department of Textile Engineering, Chemistry, and Science, North Carolina State University, 1020 Main Campus Drive, Raleigh, NC 27606 USA

**Keywords:** Formate dehydrogenase, Enzyme production, Industrial biotechnology, Techno-economic analysis, Enzyme catalysis, Carbon dioxide utilization, C1 bioeconomy

## Abstract

The conversion of carbon dioxide (CO_2_) into formate offers a promising route to enable a circular, carbon-smart bioeconomy. Formate is increasingly recognized as a versatile and energy-dense platform molecule that can serve as a feedstock for microbial fermentation, energy storage, and sustainable chemical and fuel production. A key bottleneck in this value chain is the availability of cost-effective and scalable formate dehydrogenase (FDH), which catalyze the initial reduction of CO_2_ to formate. However, little is known about the economic feasibility of producing and purifying FDH at industrial scale. In this study, we developed data-driven techno-economic models to assess the production cost of FDH in *Methylorubrum extorquens (M. extorquens)* using lab-scale data and projected outcomes across four scenarios: 1 L empirical, 5 L empirical, base, and optimistic. Our results show that the minimum selling price when using FDH as a crude protein preparation ranged from $2300/kg (1 L empirical) to $75/kg (optimistic), while the use of purified FDH resulted in costs ranging from $99,000/kg to $970/kg, respectively. Sensitivity analyses revealed that protein purity has the greatest influence on final production cost, with substrate and electricity costs also contributing significantly to the two empirical scenarios. These findings provide insight into cost bottlenecks and help identify engineering targets for scaling FDH enzyme production, supporting the development of CO_2_-to-formate technologies and the broader formate-based bioeconomy.

**Graphical abstract:**

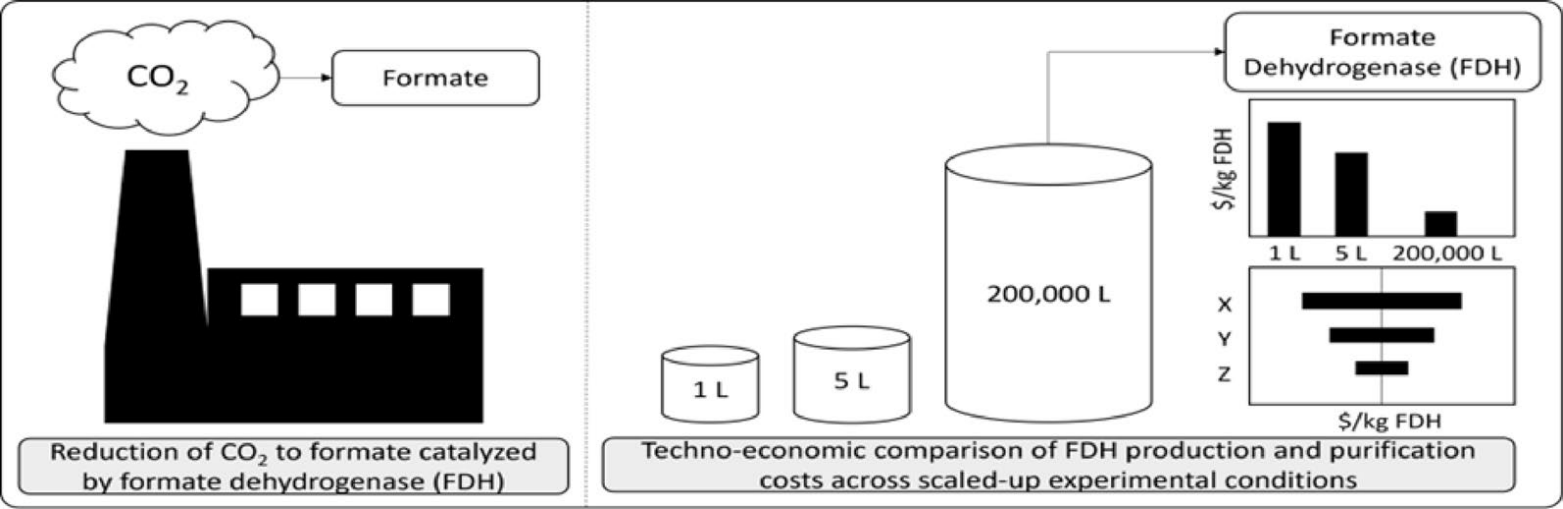

## Introduction

To meet the United States’ net-zero emissions goal by 2050, technologies that reduce greenhouse gas (GHG) emissions, particularly carbon dioxide (CO_2_), are essential. One promising approach is the enzymatic conversion of CO_2_ into value-added chemicals and fuels using redox enzymes that drive electron transfer reactions (Bierbaumer et al. 2023; Iliuta and Larachi 2023). Techno-economic analyses (TEAs) help identify cost- and energy-intensive steps in these pathways. While small-scale experimental data improve TEA accuracy, there remains a key gap in understanding the industrial-scale production costs of redox enzymes (Klein-Marcuschamer et al. 2012).

Redox enzymes, as oxidoreductases, catalyze reactions central to respiration and photosynthesis and are increasingly relevant in engineered carbon conversion systems (Léger and Bertrand 2008). A leading candidate is formate hydrogenase (FDH), which catalyzes the reversible conversion between formate and CO_2_. In nature, FDH supports microbial respiration by oxidizing formate to CO_2_ and releasing reducing equivalents (Calzadiaz-Ramirez and Meyer 2022). While the reverse reaction—reducing CO_2_ to formate—is less favorable in natural systems, it becomes viable under engineered anaerobic conditions, enabling carbon capture and conversion into soluble, energy-dense compounds like formate or formic acid.

FDH catalyzes HCOO^−^ ↔ CO_2_ + H^+^ + 2e^−^ and the reverse (CO_2_ + H^+^ + 2e^−^ ↔ HCOO^−^), using cofactors such as nicotinamide adenine dinucleotide (NAD +) or nicotinamide adenine dinucleotide phosphate (NADP^+^) for oxidation and NADH or NADPH for reduction. However, its oxygen sensitivity limits FDH activity and stability during both production and application (Cha et al. 2023; Fasano et al. 2024), presenting a significant barrier to industrial deployment.

Recent advances in genetic engineering have enabled heterologous expression of FDH in microbial hosts. For instance, expression in *Picochlorum renovo* enabled formate-based growth of the microalga (Dahlin et al. 2023), and structural studies of recombinant FDH from *M. extorquens* (formerly *Methylobacterium extorquens*) have revealed active-site features that support enzyme optimization (Park et al. 2024). Despite such progress, the scalability and cost of FDH production at industrial levels remain largely unexplored.

Enzyme production strategies vary with application, particularly with regard to purity. Pharmaceutical enzymes require high purity at low volumes, while industrial enzymes for food, textiles, and biofuels are produced in bulk at lower purities (Headon and Walsh 1994). However, rising use of immobilized enzymes is increasing purity demands, as impurities can reduce catalytic stability, binding efficiency, and overall performance (Cao 2005).

Given evolving demands and economic constraints, integrated strategies that combine biological and chemical approaches have emerged as a promising route for enzyme production. Biologically, advances in genetic engineering and microbial expression systems enable scalable and efficient enzyme synthesis (Huang 2024). Chemically, post-production modifications can enhance stability, catalytic activity, or cofactor compatibility without the need for extensive genetic redesign (Minten et al. 2014). This hybrid approach reduces production costs by improving enzyme performance and operational longevity, thereby decreasing the frequency of replacement and the burden on downstream processing. Compared to purely biological or chemical methods, this integration offers a more balanced and flexible framework for optimizing enzyme characteristics while maintaining industrial feasibility (Farhan et al. 2025; Wahab 2025).

Enzyme production generally includes follows four stages: synthesis, recovery, purification, and formulation. In synthesis, microbial cells are cultivated—usually via fermentation—to produce intracellular or extracellular enzymes (Illanes 2008). Genetic engineering can optimize secretion pathways to enhance yields (Becerra et al. 1997). Recovery methods depend on enzyme location: extracellular enzymes are recovered via centrifugation or filtration, while intracellular enzymes require cell disruption (e.g., sonication, homogenization, or lysis) followed by solid–liquid separation (Illanes 2008).

Purification removes contaminants that may impair performance, especially in pharmaceutical or immobilized enzyme applications (Remans et al. 2022). While it improves specific activity, purification reduces yield, creating a trade-off between cost and product quality (Baumann and Hubbuch 2017; Che Hussian and Leong 2024). Common purification techniques include size exclusion, ion exchange, hydrophobic interaction, and affinity chromatography (Illanes 2008). The final formulation stage stabilizes and standardizes enzymes for consistent use, ensuring batch-to-batch reliability, meeting regulatory requirements, and tailoring performance to application-specific needs (Illanes 2008).

Although redox enzyme TEAs are limited, a few studies provide cost benchmarks. Walwyn et al. (2015) estimated the cost of producing horseradish peroxidase at ~ $1280/g ($1.28 million/kg) at a small-scale capacity of 5 kg/year. Market prices corroborated these figures at $1250/g ($1.25 million/kg), emphasizing the high cost of redox enzymes at limited production scales. However, enzyme production costs generally decline with scale (Elsner et al. 2015), reinforcing the need for TEAs that reflect industrial capacities. In contrast, hydrolases, such as β-glucosidase, have been extensively studied due to their low cost and operational robustness. Ferreira et al. (2018) estimated β-glucosidase production at $316/kg for a large-scale operation producing 88,000 kg annually. This stark difference highlights the need for innovation and optimization in redox enzyme manufacturing to make them economically competitive.

Commercially available FDH remains costly and exhibits modest activity. For example, FDH from *Candida boidinii* (5–15 U/mg) is sold at $1670 per 500 units, whereas laccase—another redox enzyme—costs $199 per 10,000 units (~ 50 U/mg) (Millipore Sigma 2025a; Millipore Sigma 2025b). Despite rising interest in enzymatic CO_2_ conversion, limited empirical data exist for TEAs of redox enzyme manufacturing. This study incorporates laboratory-scale FDH production data into a TEA, offering grounded cost projections and identifying process bottlenecks. These insights provide a foundation for improving the economic viability and scalability of FDH-based CO_2_ utilization technologies.

## Methodology

### Modeling and simulation software

**Fig. 1:**
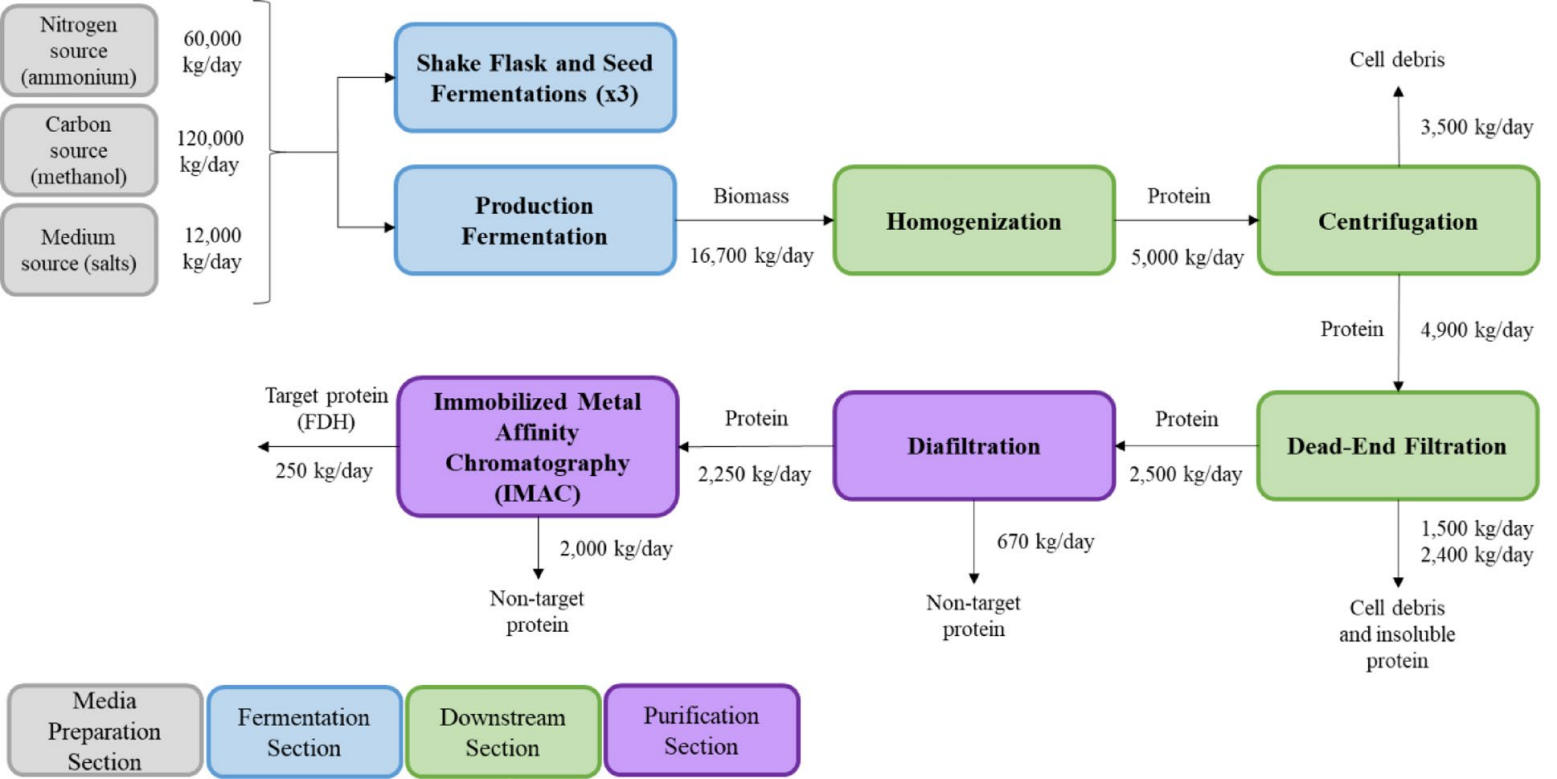
Process flow diagram of the base case FDH production and purification process with mass flows

SuperPro Designer v13 (Intelligen, USA) was used to model and simulate the process, which contained four main sections: media preparation, fermentation, downstream processing, and purification, as shown in Fig. 1. Additionally, data-driven stoichiometric reactions were established for the fermentation and parts of the downstream processes to determine the amounts of both reactants and products utilized and produced, respectively. Microsoft Excel was used to assess the economic feasibility of the process, taking into account the various capital and operating costs.

### Process and parameters

For the laboratory experiments, the alpha and beta subunits of the formate dehydrogenase enzyme MeFdh1 from *Methylorubrum extorquens* AM1 were cloned into an expression vector to enable recombinant protein production. A 6 × histidine tag was fused to the C-terminus of the alpha subunit to facilitate protein purification. The plasmid backbone, pCM157 (Plasmid #45863), was obtained from Addgene. The IPTG-inducible promoter PL/O4, a strong promoter used for *M. extorquens*, was incorporated to control gene expression. Gibson assembly was used to seamlessly combine the promoter, MeFdh1 subunit genes, and plasmid backbone into the final recombinant plasmid. The plasmid was introduced into *M. extorquens* AM1 via electroporation to construct a recombinant strain. Electro-competent cells were prepared by growing *M. extorquens* AM1 to exponential phase (OD600 0.6–0.8), chilling the culture, and washing the cells with ice-cold sterile distilled water and 10% glycerol. The cells were then suspended in 10% glycerol and mixed with 2 µl of plasmid DNA in a chilled cuvette. Electroporation was performed using a Gene Pulser (Bio-Rad) with settings of 2.50 kV, 400 Ω, and 25 µF for a 2-mm gap cuvette. Post-electroporation, Nutrient Broth was added, and the cells were incubated at 30 °C for 12 h with 200 rpm shaking. Transformants were selected on succinate agar plates containing appropriate antibiotic (kanamycin), with colonies appearing after approximately 3 days (Carrillo et al. 2019).

Inoculum was prepared from a single colony in media containing succinate or methanol, allowing them to grow overnight at 30 °C until the optical density (OD) exceeded 0.7. Protein expression was then induced using isopropyl β-D-thiogalactopyranoside (IPTG) while maintaining the temperature at 30 °C. After induction, the fermentation was carried out at 30 °C, pH 7.0, and 180 rpm in a New Brunswick Bioflo fermenter (New Brunswick Scientific, Edison, NJ, USA) with a total volume of 2.3 L and a working volume of 1 L. Various parameters were recorded, such as biomass cell density and total target protein, to incorporate into our TEA. Cell lysis of the 1 L shaking flask cell pellets was performed via sonication (60–80% amplitude, 30 s intervals, 5 min) using a FisherBrand Model 120 Sonic Dismembrator (Thermo Fisher Scientific, Waltham, MA, USA). 5 L fermentation cell pellet lysis was completed using a Niro Soavi Type NS1001 High Pressure Homogenizer. MeFDH1 purification by immobilized metal affinity chromatography (IMAC) was performed on a BIO-RAD NGC Quest 10 Plus FPLC with a 5 mL Cytiva HisTrap HP column (Park et al. 2024).

In our data-driven models, the annual enzyme production rate was 80 tonnes of enzyme/year on a dry basis for the purified enzyme cases. The media preparation section included nitrogen (ammonium), carbon (methanol), and medium (salts) sources, each linked to a 10 L shake flask, three seed fermentors with capacities of 100 L, 1000 L, and 10,000 L, and a 200,000 L production-scale fed-batch fermentor. Since *Methylorubrum extorquens* is a strictly aerobic bacterium, compressed air was injected into each fermentor to maintain an aerobic environment (Peyraud et al. 2011). Following the production fermentor stage was the product recovery or downstream section that included a homogenizer, centrifuge, and dead-end filter (Ferreira et al. 2018). The soluble protein underwent two purification steps, while the insoluble protein was considered a solid byproduct that could be sold for revenue. After purification via diafiltration and IMAC, a pure stream of FDH was produced. Figure 1 presents the process flow diagram with mass flows for the base case. The production of crude protein was also simulated to evaluate the associated protein costs, where the process for these scenarios excluded both diafiltration and IMAC unit operations.

In this study, SuperPro models were developed based on laboratory-scale fermentation data from 1 and 5 L vessels, which served as the empirical cases. Assumptions regarding biomass composition, including protein content and distribution, were made using values from the literature and experimental observations to support the techno-economic analysis. To evaluate potential improvements in process performance, two additional model scenarios—designated as the base and optimist cases—were incorporated to represent forward-looking projections. These scenarios reflect varying levels of process maturity and were designed to capture a range of feasible outcomes, from current experimental conditions to idealized industrial performance.

The empirical case represents observed laboratory-scale performance without assuming significant optimization and serves as a realistic lower bound for techno-economic feasibility. The base case incorporates moderate, near-term improvements considered achievable though standard process refinements, such as enhanced expression system stability and optimized media formations. The optimistic case reflects high-performance outcomes informed by industrial benchmarks and literature reports, serving as a proxy for a fully optimized industrial process. This is supported by Ferreira et al. (2018), who demonstrated that protein expression levels of 1–10% of cell dry weight (2–20% of total protein) are attainable in *Escherichia coli* under industrially optimized conditions.

While the optimistic case explores the upper bounds of what may be achievable, it still includes simplifications relative to real-world industrial practice—for example, assumptions of consistent upstream performance, ideal batch utilization, and simplified downstream recovery. These abstractions were necessary to maintain model transparency and comparability across scenarios, allowing the analysis to focus on identifying the key performance levers that most strongly influence economic viability. The specific values used for the empirical, base, and optimistic cases are summarized in Table 1.

**Table 1:** Process parameters for empirical, base, and optimistic cases for FDH enzyme

Model case	Biomass cell density (g/L)	Protein (% of cell)	Soluble protein (%)	Target protein (%)
Empirical (1 L)	4.2	30	3.6% of cell (12% of total protein)	0.1% of cell (0.3% of total protein)
Empirical (5 L)	6.5	30	12.6% of cell (42% of total protein)	0.6% of cell (2% of total protein)
Base	20	30	15% of cell (50% of total protein)	1.5% of cell (5% of total protein)
Optimistic	50	30	15% of cell (50% of total protein)	5% of cell (17% of total protein)

Although our models focused on a single industrial-scale capacity, the design accounted for typical scale-up considerations encountered in real-world production. As production throughout increases, downstream operations—such as centrifugation, filtration, and chromatography—can become significant bottlenecks due to volume limitations and extended processing times. Addressing these constraints may require strategies such as parallelization (deploying multiple units concurrently) or process intensification through continuous operation or the implementation of higher-capacity equipment. In addition, utility demands, such as water, steam, and electricity, may increase disproportionately with scale, impacting both capital infrastructure and operational efficiency. Our model incorporates these considerations by adjusting equipment appropriately and applying realistic assumptions for process throughput and operation times to reflect industrial-scale feasibility. To evaluate the impact of uncertainties, a sensitivity analysis was conducted on key cost and performance parameters, including protein purity, substrate and electricity costs, and downstream bottlenecks. This analysis helped identify the most influential variables on the cost of FDH production and provided insight into how process configurations may need to adapt at scale to maintain economic viability.

### Economic assumptions

When developing the industrial-scale TEA from laboratory-scale data, several assumptions were made. Table 2 outlines the economic assumptions and their respective values used in the model for the base case. An annual enzyme capacity of 80 tonnes of enzyme/year was chosen to align closely with the 88 tonnes of enzyme/year, as reported by Ferreira et al. (2018), allowing for an accurate representation of our TEA on an industrial scale. To ensure comparability across scenarios, this consistent capacity value was specified for all pure enzyme cases. Economic parameters, such as equipment sizing, capital and operating costs, and raw material consumption, were adjusted to reflect this commercial production scale. Economies of scale were incorporated to account for reductions in unit production cost with increasing throughput (Elsner et al. 2015). Additionally, the study by Ferreira et al. (2018) informed assumptions in the downstream and purification sections, particularly regarding protein yield expectations and process configuration under industrial conditions.

**Table 2:** Economic assumptions used in the base case model

Parameter	Value	References
Plant utilization	90% (330 days)	Pett-Ridge et al. (2023)
Cost year	2024	
Capital cost contingency	10% of direct + indirect CAPEX	
Indirect capital cost	42.2% of total direct CAPEX	Pett-Ridge et al. (2023)
Fixed operating cost	4.5% of total CAPEX	Pett-Ridge et al. (2023)
Return on equity	15%	
Interest rate	10%	Pett-Ridge et al. (2023)
Project life	20 years	Pett-Ridge et al. (2023)
Capital recovery factor	12.6%	
Annual enzyme capacity	80 tonnes enzyme/year	Ferreira et al. (2018)

CAPEX, capital costs; OPEX, operating costs

## Results and discussion

### Economic assessment

We estimated the levelized costs of both crude and pure protein for the empirical, base, and optimistic cases, using the process parameters and assumptions outlined in Tables 1 and 2. Based on the 1 L empirical results, the specific activities were 0.56 U/mg for the crude protein and 0.89 U/mg for the purified protein. In comparison, the 5 L experiments yielded specific activities of 0.11 U/mg for the crude protein and 1.98 U/mg for the purified protein. The enzyme unit, U, represents the enzyme activity that converts 1 micromole of substrate into products per minute (Ibrahim et al. 2021). For both the base and optimistic scenarios, the activity levels measured in the 5 L experiments were used, as this scale better reflects semi-pilot production conditions and tends to provide more stable and representative activity data. In comparison, the 1 L experiments represent an earlier stage with greater variability and less predictive value for larger-scale processes. As exhibited in Table 3, there was a wide range of capacities and protein costs across the cases.

**Table 3:** Summary of TEAs for empirical, base, and optimistic cases

Model case	Empirical (1 L)		Empirical (5 L)		Base		Optimistic	
Biomass cell density (g/L)	4.2	4.2	6.5	6.5	20	20	50	50
Target protein (% of cell)	–	0.1	–	0.6	–	1.5	–	5
Crude or pure protein	Crude	Pure	Crude	Pure	Crude	Pure	Crude	Pure
Specific activity (U/mg)	0.56	0.89	0.11	1.98	0.11	1.98	0.11	1.98
Capacity (kg protein/year)	2.9 M	80 k	1.7 M	80 k	800 k	80 k	240 k	80 k
Total Levelized Cost ($/kg protein)	2300	99,000	500	16,000	120	3600	75	970
Total Levelized Cost ($/U)	4.05E–3	1.11E−1	4.56E−3	8.23E−3	1.05E−3	1.80E−3	6.82E−4	4.88E−4

U is the enzyme activity that transforms 1 micromole of substrate (i.e., CO_2_) into products (i.e., formate) per minute under specified conditions (Ibrahim et al. 2021)

All process scenarios in this analysis were designed to deliver 80,000 kg of pure protein per year, either directly or through sufficient production of crude protein to meet this purified yield after downstream processing. Despite this consistent output target, substantial variation was observed in both levelized protein costs and specific process performance metrics due to differences in upstream and downstream assumptions. A clear inverse relationship was observed between levelized protein cost and two upstream parameters: biomass cell density and target protein content. In the empirical scenarios, the 1 L fermentation yielded a biomass density of 4.2 g/L with a target protein content of 0.1% of cell mass, resulting in a levelized pure protein cost of approximately $99,000/kg. The 5 L case showed moderate improvement, with a biomass density of 6.5 g/L and target protein content of 0.6% leading to reduced, but still high, production costs. In contrast, the base and optimistic scenarios assumed significantly higher biomass concentrations (20–50 g/L) and increased protein expression levels (1.5–5% of cell mass). These improvements resulted in substantially lower crude and pure protein costs. For example, in the optimistic case, a cell density of 50 g/L and target protein content of 5% yielded a pure protein cost of $970/kg—a more than 100-fold reduction compared to the 1 L case. This trend can be attributed to the fact that higher biomass densities reduce the required fermentation volume and associated fixed costs, while higher protein expression increases the proportion of biomass contributing to the final product. These improvements also ease downstream processing, as cell lysis and protein purification are more efficient when product titers are high.

An unusual observation was made in the empirical 1 L crude case, where the model projected a very high annual crude protein production capacity (2.9 million kg), yet the levelized cost remained high at $2300/kg. This counterintuitive result can be explained by the extremely low biomass cell density in this case (4.2 g/L), which required processing exceptionally large fermentation volumes to reach the target output. The dilute broth led to high capital costs for oversized fermenters and increased operating costs associated with media, utilities, and downstream processing. In this context, the large-scale handling of low-density biomass imposes a significant economic burden, and the potential benefits of economies of scale (Elsner et al. 2015) were insufficient to offset these inefficiencies. Although the modeled capacity was high in mass terms, the process was fundamentally constrained by the cost structure imposed by dilute feedstocks, resulting in elevated unit costs.

Crude protein consistently exhibited lower costs across all scenarios compared to pure protein, primarily due to increased production capacities and the exclusion of purification equipment and associated consumables. Protein purification remains a significant technical and economic challenge. Among the various purification techniques, affinity chromatography is the most widely employed due to its reliability in achieving high yield and purity at the bench scale. However, the high cost of chromatographic resins and the operational complexity of large-scale equipment limit the feasibility of chromatography for industrial applications. As a result, many companies opt to use proteins in their crude form, bypassing extensive purification steps to reduce cost and complexity in both research and manufacturing contexts (Li et al. 2024).

In the case of FDH, the trade-off between enzyme purity and production cost is particularly significant. While purified FDH offers improved specific activity, batch-to-batch consistency, and compatibility with systems such as immobilization or cofactor recycling, the additional purification steps substantially increase production costs. Conversely, crude FDH preparations are substantially more economical and may be adequate for applications where high purity is not essential—such as whole-cell catalysis (Yang et al. 2025) or low-cost carbon capture. However, crude FDH may suffer from lower activity per unit mass, batch variability, and potential interference from host cell proteins (Li et al. 2024).

TEA confirms that crude FDH production is the most cost-effective option, suggesting its suitability for downstream applications where cost is a primary constraint and enzyme purity is less critical. However, high-purity FDH may be required for more specialized uses—for instance, immobilization on electroconductive surfaces for formate production (Lee et al. 2024). In such cases, the trade-off includes not only higher costs but also potential loss of activity throughout the purification process. Park et al. (2024) reported a notable decrease in activity of the tungsten-containing, oxygen-tolerant MeFDH1 following a three-step purification workflow, despite its otherwise favorable performance under aerobic conditions.

These trade-offs underscore the importance of aligning enzyme production strategies with the specific requirements of the intended application. Rather than defaulting to high-purity FDH, producers can optimize costs by tailoring purification levels based on factors such as application type, production scale, required enzyme stability, activity per unit mass, and downstream integration (e.g., immobilization or cofactor recycling). These variables directly shape the techno-economic landscape. In many industrial settings, a partially purified enzyme may offer the best balance between performance and cost—especially when simplified or minimal purification steps can be employed without significantly compromising function or consistency.

The cost per unit of enzyme activity ($/U) provides further insight into the tradeoffs between crude and pure protein production. Pure proteins consistently exhibited slightly higher specific activity across all scenarios—for example, 0.89 U/mg in the empirical 1 L case, compared to 0.56 U/mg for the corresponding crude extract. In the 5 L empirical case, specific activity was 1.98 U/mg for pure protein and 0.11 U/mg for crude. However, these gains in activity came with increased $/U values, reflecting the higher $/kg production costs of purified protein. Despite the increased cost, this enhanced catalytic performance can offer greater reaction efficiencies and is often required in applications that demand high specificity or biochemical precision. When accuracy and performance outweigh cost and processing speed, the use of pure protein becomes more advantageous. Conversely, when large quantities are required at minimal cost, crude preparations may be more appropriate.

These findings are consistent with previous literature. For example, Ferreira et al. (2018) reported an enzyme production cost of $316/kg for intracellular β-glucosidase at an annual capacity of 88,000 kg, using a strain expressing the target protein at 5% of cell mass. Although the production capacity in their study was comparable to our pure protein scenarios, their cost was significantly lower than the values observed in our empirical cases—primarily due to a much higher target protein expression level. In our optimistic scenario, a 5% target protein content was also assumed, which yielded a more comparable cost of $970/kg. However, this remains notably higher than the $316/kg found for β-glucosidase, as differences in purification strategies likely drive the cost gap. Our use of IMAC significantly contributes to the overall enzyme production costs. Ferreira et al. (2018) measured a specific activity of 2.3 U/mg for β-glucosidase. Although this differs from the values observed in our empirical scenarios (Table 3), differences in enzyme type and assay conditions limit direct comparison. Notably, the calculated cost per unit activity from Ferreira et al. (2018) was approximately $1.37E−4/U, which closely aligns with our activity costs for the optimistic cases ($6.82E−4/U for crude and $4.88E−4/U for pure). This comparison further underscores the critical role that upstream expression levels play in determining both protein cost and performance efficiency.

Finally, the comparison between the base and optimistic cases reveals diminishing returns with respect to further improvements in biomass density and protein expression. While the optimistic case achieves the lowest costs overall ($75/kg crude, $970/kg pure), the cost reduction relative to the base case is modest compared to the substantial improvements observed when transitioning from empirical to base scenarios. This suggests that beyond a certain point, additional upstream optimization yields progressively smaller economic benefits, likely due to downstream constraints such as purification yield, material recovery efficiency, and consumable costs, which begin to dominate the overall cost structure.

In summary, protein production costs were strongly influenced by upstream parameters such as biomass density and protein expression, which reduce downstream burden and enable lower levelized costs. However, scale and process integration play equally important roles, as even large-scale protein output is not economically viable if achieved through inefficient, dilute processes. Moreover, the choice between crude and pure protein should be guided by application-specific requirements, balancing cost, complexity, and performance. These results emphasize the importance of aligning process design with both technical constraints and end-use demands when evaluating the economic feasibility of enzyme manufacturing systems.

To better understand how these dynamics manifest at the process level, the specific methods used were examined in our protein production pipeline and their associated cost implications. The described methods significantly influence the overall cost model in a TEA of protein production. Starting from a single colony and growing the cells in media containing succinate or methanol ensures consistency and reproducibility, though the choice of carbon source can impact media costs and growth efficiency. Protein expression was induced using IPTG, a potent but costly inducer that increases upstream processing expenses. Fermentation adds operational costs related to temperature control, agitation, and equipment usage. As previously mentioned, parameters including biomass cell density and total target protein were noted and incorporated into our TEA, enabling a more accurate assessment of process efficiency and yield. Cell lysis methods also contribute to cost variation—sonication is suitable for small volumes but is energy-intensive and laborious, while high-pressure homogenization is more scalable but requires expensive equipment. Downstream, the use of IMAC is highly effective but represents a major cost driver in purification. Together, these upstream and downstream steps determine the overall productivity and economic viability of the process.

Table 4 further analyzes the cost comparisons between the two products, outlining the various capital and operating cost contributions to the base case levelized costs of the crude and pure protein. In both cases, the methanol feed rate is the same at 120,000 kg per day.

**Table 4:** Summary of base case TEAs for the production of crude and pure proteins

	Crude protein	Pure protein
Annual protein capacity	800,000 kg protein/year	80,000 kg protein/year
Methanol feed rate	120,000 kg/day	120,000 kg/day
Total capital cost	$118 M	$268 M
Levelized capital cost	$21/kg protein	$467/kg protein
% of total levelized cost	17.78%	13.07%
Total operating cost	$77 M/year	$250 M/year
Net operating cost	$76 M/year	$248 M/year
Levelized operating cost	$95/kg protein	$3103/kg protein
% of total levelized cost	82.22%	86.93%
Total levelized cost	$120/kg protein	$3600/kg protein

The base case TEA highlights significant cost differences between crude and pure protein production, driven largely by differences in downstream processing requirements, capital intensity, and achievable scale. First, the annual protein capacity differs by an order of magnitude: 800,000 kg/year for crude protein versus only 80,000 kg/year for pure protein. This disparity is not due to differences in upstream fermentation or feedstock input—both scenarios assume the same methanol feed rate of 120,000 kg/day—but instead reflects the additional burden of purification, which limits the amount of product that can be feasibly recovered and processed.

The lower throughput of the pure protein scenario amplifies both capital and operating costs on a per-unit basis. The total capital cost for pure protein production is more than double that of the crude case ($268 million vs. $118 million), despite the lower product output. As a result, the levelized capital cost per kilogram of protein is over 20 times higher for pure protein ($467/kg vs. $21/kg). However, capital costs account for a smaller fraction of the total levelized cost in both cases (17.78% for crude, 13.07% for pure), indicating that operating costs are the dominant economic factor.

Operating costs provide further evidence of this trend. While both systems operate with similar feed input rates, the total and net operating costs for pure protein production are over three times greater than for crude protein ($250 million/year vs. $77 million/year total operating; $248 million/year vs. $76 million/year net operating). This is primarily due to the intensive requirements of protein purification, including multiple chromatography steps, consumables, and additional buffer volumes. As a result, the levelized operating cost per kilogram is $3103/kg for pure protein compared to only $95/kg for crude protein. Consequently, the total levelized cost of pure protein production is dramatically higher—$3600/kg compared to $120/kg for crude protein—underscoring the cost challenges associated with achieving high-purity protein at scale.

Analysis of the data reinforces purification as the principal economic bottleneck in enzyme manufacturing. While capital cost differences are notable, the purification burden primarily manifests in ongoing operating expenses. Therefore, for applications where crude protein is functionally sufficient, the cost savings are substantial and could justify the trade-off in product quality or purity. This is exemplified in biofuel production, where enzymes—particularly cellulases—are typically used in crude form and secreted extracellularly (Gao et al. 2015), making them more feasible and cost-effective than intracellular enzymes, such as FDH, which require additional extraction and purification steps. However, the use of crude enzymes is not without drawbacks. For instance, in crude FDH preparations, inhibitors or interfering compounds such as residual peptide tags on the recombinant protein, nucleic acids, and non-target proteins (Rosano and Ceccarelli 2014) may be present, potentially reducing enzyme activity (Cao 2005). Moreover, due to the limited empirical data available in the literature, it remains too early to determine the extent to which these impurities affect performance. Conversely, in applications that demand high specificity, selectivity, or adherence to regulatory standards, the addition expense of producing purified protein may be warranted—provided that the downstream value and performance benefits justify the higher production costs.

To assess the influence of various factors, Table 5 presents a comparison of enzyme production parameters from multiple studies, emphasizing the range of costs, expression systems, and production efficiencies across different enzymes. This comparison offers critical insights into the diverse factors affecting enzyme production processes and elucidates the key determinants of cost and operational efficiency in different industrial contexts.

**Table 5:** Comparison of reported parameters from previous studies with those in the current study

Specific enzyme	Enzyme class	Enzyme expression	Cell density	Target protein percentage	Protein production capacity	Fermentation scale	Purification method(s)	Capital cost	Operating cost	Enzyme cost ($/kg)	Enzyme cost ($/U)	References
FDH (base case)	Redox	Intracellular	20 g/L	1.5% of cell	80,000 kg/year	200,000 L	Diafiltration, IMAC	$268 million	$248 million/year	$3600/kg	$1.80E−3/U	This study
Horseradish Peroxidase	Redox	–	–	–	5 kg/year	–	Ion exchange chromatography, Ultrafiltration	–	–	$1280,000/kg	–	Walwyn et al. (2015)
β-glucosidase	Hydrolase	Intracellular	100 g/L	5% of cell	88,000 kg/year	100,000 L	Diafiltration	$70.8 million	$27.9 million/year	$316/kg	$1.37E−4/U	Ferreira et al. (2018)
Protease	Hydrolase	Extracellular	–	–	30,600 kg/year	11,500 L	–	$308,000	$24,300/year	$2.12/kg	$8.83E−7/U	Rao et al. (2017)
Lipase	Hydrolase	Extracellular	–	–	605 kg/year	40 L	Packed bed absorption	$264,000	$2.66 million/year	$4400/kg	$5.81E−3/U	Kumar et al. (2023)
Lipase	Hydrolase	Extracellular	–	–	4290 kg/year	–	–	$302,000	$123,000/year	$65/kg	–	Khoomata et al. (2018)
Cellulase	Hydrolase	–	–	–	5.7 million kg/year	–	Used, but not specified	$18 million	–	$3.80–$8.80/kg	–	Hong et al. (2013)
Cellulase	Hydrolase	–	–	–	525,000–758,000 kg/year	938,000–2,740,000 L	–	$22.0–$28.6 million	$8.23–$30.6 million/year	$15.67–$40.36/kg	–	Zhuang et al. (2007)
Cellulase, Endo-β-1,4-glucanase, Laccase	Hydrolase	Extracellular	–	–	–	–	–	–	–	$21–$42/kg $250/kg $14/kg	–	Sosa-Martínez et al. (2024)
Enzyme Cocktail	Hydrolase	Extracellular	–	–	5000 kg/batch (35–704 batches/year)	8030 L	Diafiltration	$4.5 million	$3.3 million/year	$1.92–$3.72/kg	–	Sosa-Martínez et al. (2024)

U is the enzyme activity that transforms 1 micromole of substrate (i.e., CO_2_) into products (i.e., formate) per minute under specified conditions (Ibrahim et al. 2021)

As shown in Table 5, TEAs of enzyme production demonstrate significant variability in cost estimates depending on scale, purification methods, and enzyme type. For example, Kumar et al. (2023) estimated lipase production at $4400/kg using a small-scale (40 L) reactor and packed bed absorption, while our model, using a 200,000 L fermentor and two-step purification, yielded a lower cost of $3600/kg due to economies of scale and higher throughput (80,000 kg/year versus 605 kg/year). Other studies, such as those by Hong et al. (2013) and Zhuang et al. (2007), reported cellulase costs ranging from $3.8/kg to $40/kg at much higher scales (up to 5.7 million kg/year), though often with less intensive purification or without specifying purification methods, which likely contributed to their lower reported costs.

Lipase and protease production costs estimated by Khootama et al. (2018) and Rao et al. (2017) were significantly lower than in our study, largely due to smaller scale operations without purification steps. Sosa-Martinez et al. (2024) also showed lower enzyme costs ($2-$250/kg) due to minimal purification and extracellular enzyme secretion. However, differences in purification methods—such as the use of IMAC in our study—drove up costs substantially, with IMAC alone contributing over 30% of total direct capital costs. In contrast, the TEA by Walwyn et al. (2015) reported horseradish peroxidase costs at $1.28 million/kg due to extremely small scale (5 kg/year) and costly downstream processing, illustrating how economies of scale and purification strategy are critical to cost efficiency in enzyme manufacturing. Table 5 highlights that Walwyn et al. (2015) was the only study focusing on redox enzymes, revealing a gap in TEAs for these enzymes, such as FDH, suggesting that these bioprocesses need further cost optimization.

### Sensitivity analysis

**Fig. 2:**
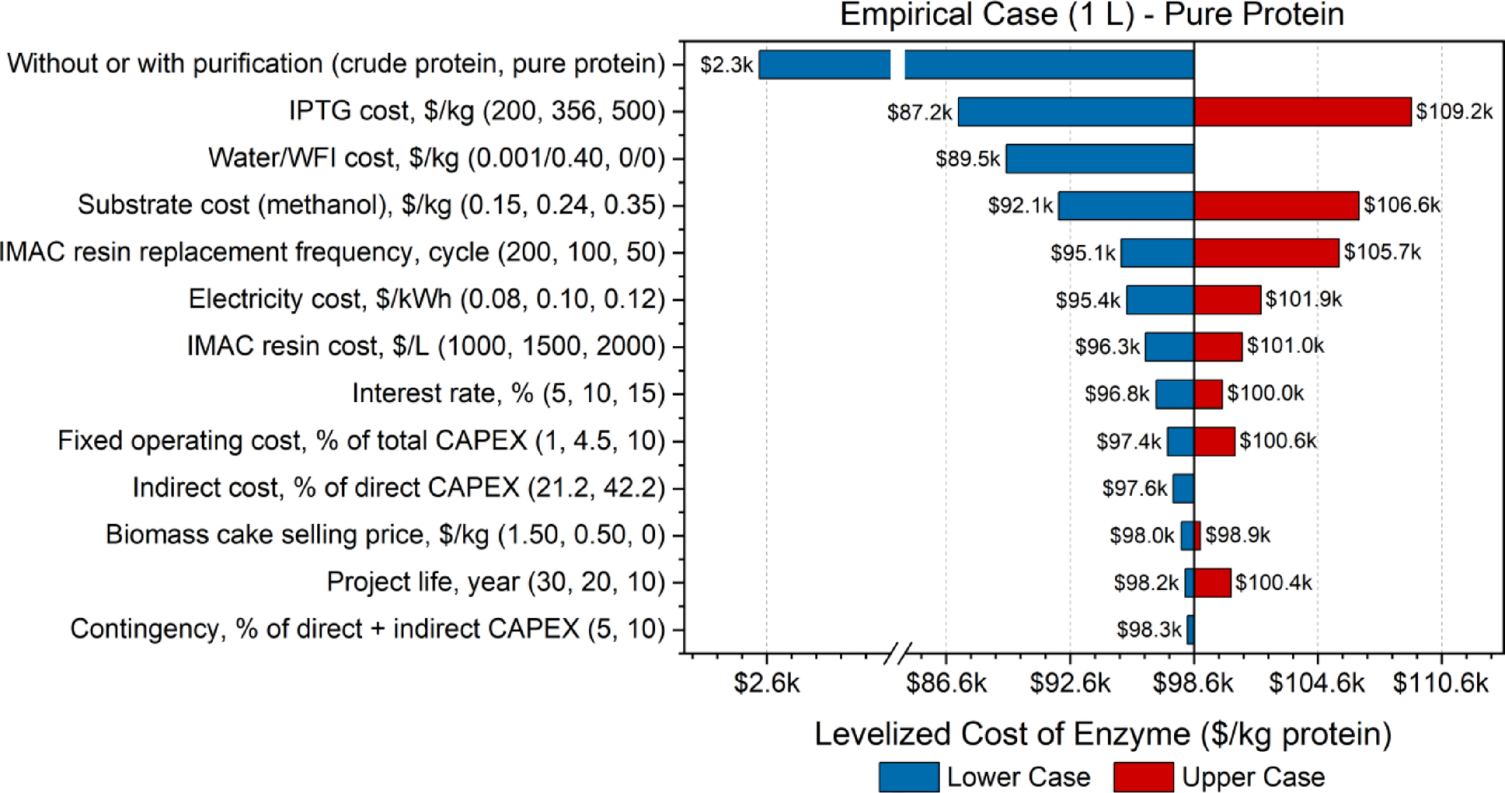
Tornado plot displaying the sensitivity analysis of pure protein 1 L empirical case

**Fig. 3:**
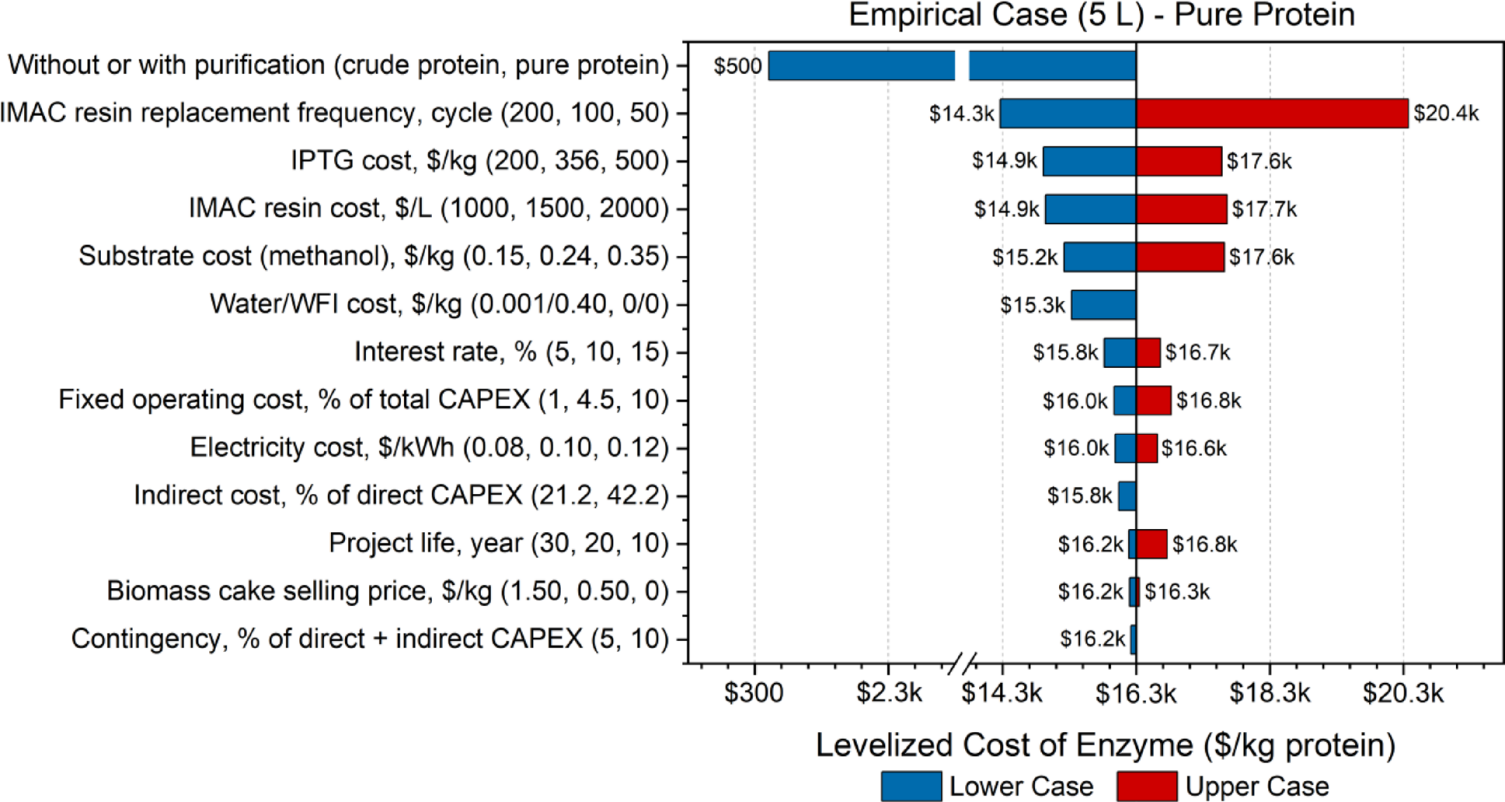
Tornado plot displaying the sensitivity analysis of pure protein 5 L empirical case

**Fig. 4:**
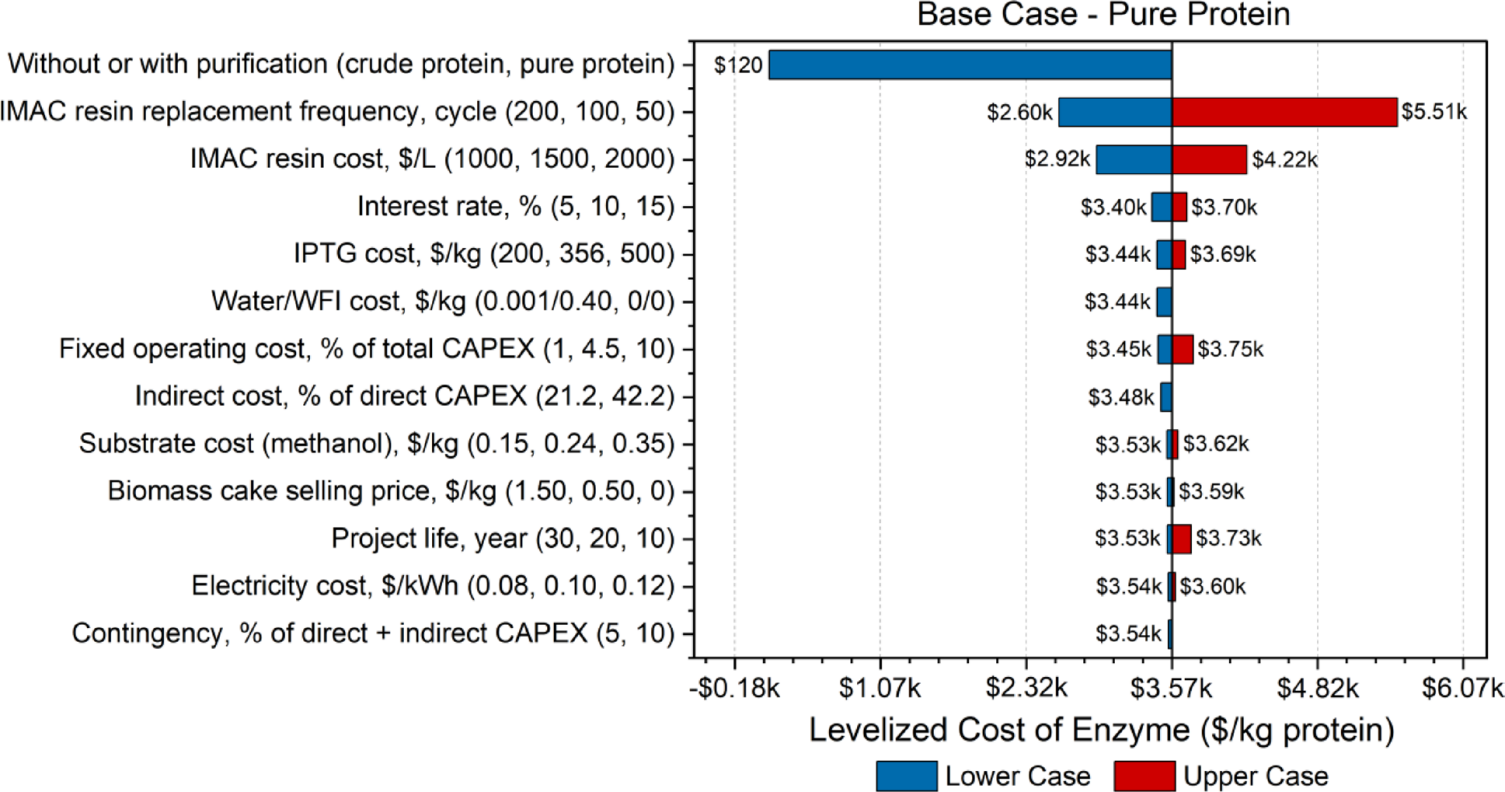
Tornado plot displaying the sensitivity analysis of pure protein base case

**Fig. 5:**
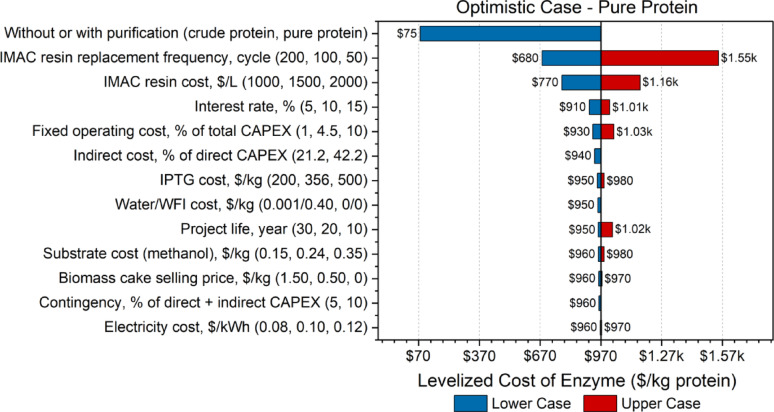
Tornado plot displaying the sensitivity analysis of pure protein optimistic case

Sensitivity analyses evaluate the impact of variations in key variables or input parameters on the overall economic performance of the process. Given that this study focused on an early-stage experimental FDH production process, there was inherent uncertainty across various stages. By testing and modifying specific parameter values, researchers can identify optimal ranges that minimize enzyme costs. In the sensitivity analyses, shown in Figs. 2, 3, 4 and 5, the effect of changes in different parameters are visually evident in the form of a tornado plot. The x-axis represents the enzyme selling price in USD per kg protein, while the y-axis specifies the parameters tested. For each parameter, the first value listed is the lower case, the second value is the base case for each particular model, and the third value is the upper case.

The sensitivity analysis presented in Fig. 2 for the 1 L empirical case highlighted the critical influence of specific process variables on the final enzyme production costs. One of the most impactful parameters was the extent of protein purification. The analysis showed that the cost of crude protein was about $2300/kg, whereas the cost of purified protein rose sharply to nearly $99,000/kg, corresponding to an increase of over 4000%. This stark contrast reflects both the reduction in overall yield during purification and the significant material and equipment costs associated with the purification steps. For instance, the diafiltration membrane and IMAC resin accounted for 8.6% and 7.6% of total variable operating costs, respectively, in the purified protein scenario. Additionally, IMAC resin and associated equipment constituted about 12.6% of the total direct capital cost. These values were derived from process cost modeling using itemized equipment and consumable prices, linked to the mass flow and equipment usage data from the empirical runs. When these two purification steps were removed in the crude protein scenario, the total production cost dropped substantially, clearly quantifying their economic impact.

Another highly sensitive input was the cost of the inducer isopropyl β-D-thiogalactopyranoside (IPTG), which was found to alter the enzyme cost by approximately $10,000 across the tested range. IPTG alone contributed 28.2% of the total viable operating cost, highlighting its outsized influence despite its small mass input. Cost sensitivity was quantified by varying IPTG price within realistic market bounds and observing the resulting shift in total production cost. To address this, lactose was identified as a potential low-cost alternative ($2/kg compared to $356/kg for IPTG), which could substantially reduce induction costs (Xu et al. 2023). However, recent advances in synthetic biology, including the availability of synthetic genes and engineering strains (Chang et al. 2024), are enabling alternative induction systems that may further optimize enzyme production, potentially reducing reliance on lactose in some applications.

Other cost-sensitive parameters included water and substrate costs, IMAC resin replacement frequency, and electricity. For example, increasing substrate cost by around $0.10/kg resulted in an 8.1% increase in overall enzyme cost. These relationships were quantified by varying each parameter individually within literature-informed ranges and analyzing their specific impact on total cost. The rest of the parameters tested were not significantly sensitive, demonstrating that changes in these parameters should not have a large impact on the enzyme price. This quantification approach allowed clear prioritization of cost drivers, helping to identify which variables warrant optimization in future scale-up scenarios.

Consistent with the 1 L empirical case, protein purification remained the dominant cost driver in the 5 L empirical model (Fig. 3). The cost of crude protein was about $500/kg, whereas purified protein reached $16,000/kg, representing an approximate 3000% increase. This substantial rise resulted from the added cost of purification materials and reduced overall protein yield. Among the most influential variables, the IMAC resin replacement frequency, as well as the costs of IPTG, methanol, water, and the IMAC resin itself, exhibited notable sensitivity. For instance, IPTG and IMAC resin contributed 21.8% and 27.9% to the total variable operating costs, respectively, indicating that even modest fluctuations in their prices and usage rates can meaningfully alter the final enzyme cost. Methanol accounted for 19.8% of these costs, while water contributed 6.6%, underscoring the impact of solvent usage and utility inputs. Parameters with a cost contribution below that of water generally induced changes of less than $480/kg in the final protein cost, indicating comparatively lower sensitivity. These insights inform strategic decision-making by identifying the most impactful parameters to target for reducing costs during future process scale-up.

The sensitivity analysis for the base case confirmed that the inclusion or exclusion of purification steps was the most influential factor affecting enzyme production cost, consistent with the empirical findings. As shown in Table 3 and Fig. 4, producing crude protein reduced the enzyme selling price from $3600/kg (pure protein) to $120/kg—a nearly 97% decrease—highlighting the significant cost burden imposed by purification. Similar to the 1 L and 5 L empirical models, the absence of purification and increased annual production capacity substantially lowered crude protein costs. The IMAC resin replacement frequency and cost were also highly sensitive variables. Reducing the resin lifespan from 100 to 50 cycles increased enzyme cost by $2000/kg (a 54.2% rise), whereas extending it to 200 cycles decreased the cost by about $970/kg (27.1% decrease). This sensitivity was driven by the fact that, in the base case, IMAC accounted for 55.4% of the direct capital costs and 65.2% of the variable operating costs.

To mitigate these cost drivers, future research could focus on expressing active FDH extracellularly, enabling recovery via filtration and eliminating the need for IMAC-based purification altogether. Comparatively, the 1 L empirical case showed much lower sensitivity to IMAC costs, with contributions of only 12.6% to capital and 7.6% to operating costs. The 5 L model exhibited moderate sensitivity (32.9% and 27.9%, respectively), while the optimistic scenario was the most sensitive, with IMAC comprising 69.5% of capital and 76.4% of operating costs (Fig. 5). These trends demonstrate that as the economic burden of IMAC increases within a process design, resin replacement frequency becomes a more critical factor. Additionally, varying the unit price of IMAC resin by $500/L led to a $645/kg change in protein cost, further underscoring its influence. In contrast, all other parameters tested caused cost fluctuations of less than $170/kg, indicating relatively low sensitivity.

As shown in Fig. 5, the comparison between crude and purified protein production emerged as the most sensitive parameter across all scenarios. In the optimistic case, the system was particularly sensitive to changes in the IMAC resin replacement frequency. Halving the resin lifespan led to a cost increase of over $580/kg (60.1% increase), while doubling it reduced the cost by approximately $290/kg (30.0% decrease). This heightened sensitivity stems from the large proportion of IMAC-related expenses in this scenario, where the resin accounted for 76.4% of variable operating costs and a substantial portion of capital investment (69.5%). Changes in the resin’s unit price also impacted overall cost—varying by $500/L resulted in a $194/kg shift in protein production cost. By contrast, all other parameters examined produced cost changes under $60/kg, indicating a minimal effect on the final selling price.

The Ferreira et al. (2018) study, which calculated an enzyme cost of $316/kg of β-glucosidase, found that consumables, including membranes, represented 23% of the unit production cost of the enzyme. Of the raw material costs, glucose and IPTG made up 47% and 41%, respectively, while kanamycin was negligible. In our base case TEA, the membranes contributed to about 3.6% of the variable operating costs, while methanol was 3.9% and IPTG was 9.8%. The largest contributor to the operating costs was the IMAC resin, accounting for 65.2%. In contrast, the Ferreira et al. (2018) publication did not include an IMAC unit in their process, which explains why methanol and IPTG costs contributed less to the operating cost in our study. Similar to our study, the authors conducted sensitivity analyses on various parameters, where increasing the biomass density significantly reduced the resulting enzyme cost, a trend also observed in our cases with different biomass cell densities. Therefore, by maximizing the biomass density in our experiments, we could lower the enzyme costs.

Walwyn et al. (2015) conducted a TEA on the production of a redox enzyme, horseradish peroxidase, including a sensitivity analysis that highlighted protein yield (activity units/g biomass) as a key target for future research and development in this area. Additionally, increasing the production capacity by 50% significantly improved project viability, reducing the enzyme cost by $183,000/kg of peroxidase. When comparing the capacities between the crude and pure proteins, large discrepancies in the resulting costs were observed. These results emphasize the importance of optimizing both yield and scale to drive down costs and enhance the overall viability of enzyme production processes.

Another important consideration is that enzyme production processes can vary widely in structure—some produce a single target enzyme, while others yield multiple enzymes or valuable coproducts. These differences can significantly affect overall economics. In particular, raw material inputs represent a major cost component, and strategies to reduce substrate or inducer costs, such as switching from IPTG to lactose or utilizing low-cost carbon sources, can directly lower the enzyme selling price. Furthermore, scale plays a crucial role: as operational scale increases, fixed costs are distributed over a larger product volume, leading to reduced per-unit production costs. Price variability in equipment, materials, and reagents, which often depends on vendor choice and regional market conditions, also contributes to differences in total cost across systems.

Several process optimization strategies and technological advances have the potential to mitigate these costs drivers. For example, the development of production strains engineered for higher expression levels, more efficient secretion of enzymes, or tolerance to lower-cost growth media can significantly improve process economics. Synthetic biology tools, such as dynamic gene regulation, self-inducible promoters, and genome streamlining, can further reduce reliance on expensive inducers and minimize resource consumption. Likewise, upstream and downstream process integration, including high cell density fermentation and continuous purification, can enhance yield and reduce costs.

Incorporating coproduct valorization can offer additional cost offsets. Prior studies have demonstrated that residual biomass may be repurposed as animal feed or combusted for energy production in the form of steam, reducing waste disposal costs and providing auxiliary revenue streams (Ferreira et al. 2021). However, our sensitivity analysis revealed that, in this specific case, revenue from solid byproducts had a negligible impact on the final protein cost. This suggests that while coproduct utilization is context-dependent, it may not always represent a major lever for cost reduction unless carefully optimized and paired with favorable market conditions. Taken together, these findings underscore the importance of holistic process design—one that combines genetic optimization, process engineering, and system-level cost modeling—to achieve economically viable enzyme production.

In addition to technical and economic optimizations, broader external factors—such as regulatory frameworks, market dynamics, and environmental considerations—can significantly influence the feasibility and scalability of CO_2_-to-formace technologies. For example, policy mechanisms like carbon pricing, renewable fuel standards, or targeted subsidies for carbon utilization technologies could materially improve the economic outlook. These policies either incentivize CO_2_-derived products or impose costs on fossil-based alternatives, enhancing competitiveness and market entry potential (Zhang et al. 2023). Often, such policies act as critical enablers by reducing investment risks and accelerating commercialization timelines.

Similarly, growing market demand for formate as an industrial feedstock or energy carrier may shape the commercial viability of FDH-based production pathways, particularly when these are integrated within sectors actively pursuing decarbonization targets (Silveira Sbrice Pinto et al. 2025). The expanding use of formate in applications like fuel cells (Xiang et al., 2019) and chemical intermediates (Silveira Sbrice Pinto et al. 2025) underlines its strategic importance in a low-carbon economy.

Environmental performance also plays a pivotal role in shaping technology adoption. Life cycle assessments (LCAs) of CO_2_-to-formate systems provide quantitative insights into emissions reductions, resource use, and overall sustainability. These metrics are increasingly critical for securing green certifications, meeting corporate sustainability goals, and participating in emerging carbon markets (Badger et al. 2025; Paulillo et al. 2021). Demonstrating favorable environmental profiles not only supports policy compliance but also enhances stakeholder acceptance and investment attractiveness.

Taken together, these broader factors are essential for situating FDH production technologies within the evolving bioeconomy landscape. Future research would benefit from integrating these external drivers—regulatory, market, and environmental—into comprehensive scenario-based TEAs to better reflect real-world complexities and guide strategic deployment decisions.

## Conclusion

This study used SuperPro Designer to simulate industrial-scale production of formate dehydrogenase (FDH), a redox enzyme, integrating laboratory-scale experimental data into a scalable process model to estimate levelized costs for both crude and purified FDH across multiple scenarios. Empirical cases based on experimental data demonstrated high production costs, particularly for purified protein. However, modeled projections for base and optimistic scenarios—envisioned through increased biomass density and target protein expression—revealed significantly improved outcomes. In the optimistic case, costs ranged from $75/kg ($1.56E−5/U) to $970/kg ($9.21E−5/U), while the base case, which assumed moderate improvements, ranged from $120/kg ($2.40E−5/U) to $3600/kg ($3.40E−4/U). Key cost drivers included biomass cell density, protein purity, and IMAC resin replacement frequency, whereas factors like electricity cost, methanol price, interest rate, and project life had relatively minor impacts. This techno-economic framework identifies critical cost levers and guides research and development toward more efficient upstream expression, downstream purification, and scale-up of redox enzymes for next-generation biomanufacturing applications.

## Data Availability

The datasets used and/or analyzed during the current study are available from the corresponding author on reasonable request.

## Abbreviations

FDH: Formate dehydrogenase
TEA: Techno-economic analysis
